# An Account of the Effects of the Digitalis Purpurea in Dropsy

**Published:** 1785

**Authors:** 


### VII. *An account of the effects of the Digitalis purpurea in dropsy.*

WE formerly * had occasion to mention, that some trials had been made at Edinburgh with the *Digitalis purpurea*, or *Fox glove*, in dropsy. This plant, which has since been admitted into the Edinburgh Dispensatory, was given in infusion according to the following formula, viz.

R. Fol. Digital. flore purpur. ℥ij.

Aq. bullient. ℔j.

Macerentur loco tepido, vase clauso, horas sex, dein cola.

The dose of this infusion was from half an ounce to two ounces four times a day; but from a letter written by Dr. Karr, of Huntingdon, to Dr. Garthshore, and communicated to us by the latter, we now learn, that the experiments in question were not very successful. In this letter Dr. Karr, whose long residence in the Royal Infirmary at Edinburgh has enabled him to acquire the fullest information on the subject, expresses himself in the following manner:— "I am sorry to observe, that I can, from the "trials which I have seen made of the digitalis

* Vol. 2. p. 416.

" purpurea, ſay very little in its favour. For

" although I have ſeen it tried repeatedly in

" hydrothorax and other dropſies, I do not re-

" member one caſe in which it produced a

" cure. It generally, indeed, had the effect

" of increaſing the urine and diminiſhing the

" ſerous effuſion but then the ſickneſs which it

" occaſioned became ſo diſtreſſing to the pa-

" tients that they refuſed to continue the uſe

" of it."

In the courſe of laſt year a decoction of the dried plant was preſcribed by Dr. Simmons in a great number of dropſical caſes at the Weſtminſter General Diſpenſary, but without its ſeeming to be of much efficacy; having lately, however, heard that a very reſpectable phyſician at Derby had employed a decoction of the freſh plant with great ſucceſs, he determined to try the dried plant again in a larger doſe than before, not being able at this ſeaſon (the beginning of March) to procure it in a freſh ſtate. He was the more anxious to make farther trial of its efficacy, as he had the pleaſure of learning that a gentleman, who is deſervedly eſteemed as a valuable member of ſociety, had, after having long laboured under ſymptoms of hydrothorax, derived ſignal relief from a ſingle doſe of four tabletable-ſpoonfuls of a decoction, prepared by boiling four ounces of the freſh plant in a quart of water to a pint. In this doſe it produced no purgative effect, but excited an uncommon degree of nauſea, which did not entirely ſubſide for two or three days, although the medicine was not repeated; — it was very remarkable, however, that the nauſea did not begin till twelve hours after the medicine had been taken, and not till ſeveral hours after its diuretic effect, which was very great, had begun to take place.

A farther trial of the medicine was now made at the Weſtminſter Diſpenſary, by Dr. Simmons, in the caſe of a man aged fifty-two years, who was admitted at the Diſpenſary on Friday the 11th of March, 1785. He had for many years been ſubject to a cough and difficulty of breathing, which of late were much increaſed, with great quickneſs of pulſe, and other dangerous ſymptoms. His hands and feet were now, and had been for more than a fortnight, very œdematous; he was not able to lie down in his bed, and for ſome time paſt had ſeldom voided more than half a pint of urine in the ſpace of twenty-four hours.

March 12th, at ten o'clock, he took four table-fpoonfuls of a decoction, made by boiling three ounces of the dried plant in a pint and a half of water to a pint, and before noon voided a pint and a half of urine. At noon, he took, as he had been directed, two table-fpoonfuls more, and the next morning the quantity of urine he had difcharged (including the pint and a half already mentioned) exceeded four quarts.

He repeated the fame dofe of the decoction (viz. fix table-fpoonfuls) on the 13th and 14th of March, but on neither of thofe days did he void more than three pints of urine. He took it again on the 15th, when it excited naufea and vomiting, but thefe effects were neither violent nor of long continuance; and on the 16th in the evening, when this account is written, we find, that fince he took the laft dofe of medicine, yefterday, he has voided nine pints of urine. The fwelling of his hands is now wholly removed, and that of his legs and feet much diminifhed; but his other fymptoms (as might be expected in a cafe of fuch long ftanding, and in which the dropfy is merely a fecondary complaint) are ftill fuch as threaten a fatal termination. But whatever may be the event, event, the effects now related afford a ftriking proof of the diuretic power of the remedy in queftion.

It is worthy of obfervation, that in this patient the medicine has produced no purgative effect, as he has had only one ftool fince he firft began to take it. It is alfo remarkable, that, the laft dofe excepted, it has not occafioned the leaft degree of naufea, though in many patients this effect has been produced by much fmaller dofes of the fame decoction, and yet in none of thefe was its operation, as a diuretic, confiderable; and in the cafe of the gentleman alluded to in a former part of this paper, it was obferved, that the increafed flow of urine began long before the ficknefs took place.— Thefe circumftances feem to render it probable that the diuretic property of the plant does not depend altogether on its exciting ficknefs; but it muft be left to future experiments to determine whether or not, and to what degree, that property is increafed by this naufeating effect.

If it fhould appear that the ficknefs it excites does not tend to increafe the urinary fecretion, and that (as we already fufpect) this effect (viz. the naufea) depends on a virulent property of the plant, which is much diminiſhed by drying it, the dried plant will certainly be preferable to the freſh.

As ſome of our readers may be induced to try the effects of this plant in dropſies, we ſhall be thankful to them for the communication of any facts which may tend to aſcertain the degree of confidence it merits. We think it right, however, to recommend great caution in the uſe of it, particularly when the freſh plant is employed, as the *Digitalis purpurea* ranks among poiſonous plants, and may, if indiſcretely adminiſtered, produce alarming and even fatal effects.

